# Prevalence of peripheral retinal findings in retinal patients using ultra-widefield pseudocolor fundus imaging

**DOI:** 10.1038/s41598-023-47761-x

**Published:** 2023-11-22

**Authors:** Paripoorna Sharma, Ihab Shareef, Fritz Gerald P. Kalaw, Rasha Nabil Kako, Andrew Lin, Varsha Alex, Eric Nudleman, Evan H. Walker, Shyamanga Borooah

**Affiliations:** 1 University of California San Diego, La Jolla, USA; 2 Jacobs Retina Center, Shiley Eye Institute, University of California San Diego, 9415 Campus Point Drive, La Jolla, CA 92093 USA

**Keywords:** Eye diseases, Diagnostic markers

## Abstract

Ultra-widefield retinal imaging is increasingly used in ophthalmology and optometry practices to image patients identifying peripheral abnormalities. However, the clinical relevance of these peripheral retinal abnormalities is unclear. This cross-sectional study aims to firstly validate a new grading system, secondly, assess the prevalence of peripheral retinal abnormalities in retinal patients, and finally understand how peripheral findings may associate with retinal disease. Ultra-widefield pseudocolor fundus images were taken from the eyes of clinic patients. Demographic data and clinical diagnosis for each patient was noted. The grading system was validated using masked retinal specialists. Logistic regression identified associations between retinal disease and peripheral retinal findings. Using the grading system, inter-observer agreement was 76.1% with Cohen’s Kappa coefficient 0.542 (p < 0.0001) and the test–retest agreement was 95.1% with Kappa 0.677(p < 0.0001). 971 images were included, with 625 eyes (64.4%) having peripheral abnormalities. Peripheral drusen was the most common abnormality (n = 221, 22.76%) and correlated with age-related macular degeneration (p < 0.001). Novel correlations were also identified between diabetic retinopathy and retinal pigmentation as well as pigmentary degeneration. This study provides a validated system for identifying peripheral abnormalities and adds to literature highlighting peripheral retinal associations with retinal disease which would benefit from further study.

## Introduction

The use of ultra-widefield pseudocolor fundus imaging is increasing in retinal clinics^1,2^. While traditional fundus cameras are able to capture approximately 30–50° of the retina, ultra-widefield fundus imaging is able to capture a wider field of up to 200° of the retina (approximately 82%) through red (635 nm) and green (532 nm) lasers to obtain high-resolution pseudocolor images of the sensory retina to the choroid^2,3^. The increased field of view allows the rapid non-invasive imaging of the peripheral retina, which can be affected by a variety of retinal and choroidal pathology^2,3^. Currently, relatively little is known about the relationship of peripheral changes, identified on ultra-widefield imaging, with retinal disease.

Previous studies using ultra-widefield retinal imaging in normal sample populations have noted some associations with known retinal disease. Diabetic retinopathy (DR) is often associated with peripheral abnormalities including blot hemorrhages, dot hemorrhages, laser marks, and hard exudates^4–6^. Additionally, previous studies in age-related macular degeneration (AMD) patients have noted increased peripheral retinal drusen like deposits^7–9^. Other studies have also shown that posterior vitreous detachment (PVD) is associated with peripheral retinal degeneration^10^. Despite these studies, however, the presence of peripheral retinal abnormalities and their overall prevalence is still relatively underexplored in the literature and warrants further study focusing on relationships between other retinal disease and findings. Additionally, there is currently a relative lack of grading systems looking to identify peripheral retinal abnormalities^11–14^.

The present study aims to address these gaps in knowledge by validating a grading system using ultra-widefield pseudocolor imaging. This may be helpful for researchers identifying diseases associations with peripheral retinal changes. The peripheral abnormalities observed in these images will be analyzed to determine whether there are statistically significant relationships with the clinical retinal diagnoses. We will also quantify the prevalence of peripheral retinal abnormalities seen in ultra-widefield fundus pseudocolor images in retinal clinic patients. Both should be informative for physicians using retinal imaging in their clinical practice and for better understanding peripheral abnormalities associated with retinal diseases.

## Methods

The study was approved by the Institutional Review Board at the University of California–San Diego, California, USA (IRB # 120516). This study complied with the Health Insurance Portability and Accountability Act of 1996 and patient informed consent was obtained by the institution’s protocol. Data was anonymized for patient safety and all collection and analysis was conducted by the Principles of the Declaration of Helsinki.

The present study is a retrospective observational clinical study. The study included consecutive patients visiting two retinal specialists who use ultra-widefield imaging for all their patients at the Shiley Eye Institute, University of California, San Diego between the 6th of January and 29th December 2021. Each patient was first dilated with 1 drop of tropicamide 1% and phenylephrine 2.5% in each eye. After a minimum wait time of 10 min, each patient then underwent standard pseudocolor imaging (Optos P200DTx, Optos plc, Dunfermline, UK) centered at the fovea by a trained medical photographer using one camera. Only the right eye image of each patient was collected for further analysis in this study to remove potentially confounding analysis caused by the correlative effect of two eyes from the same patient. Demographic data was also collected for each patient including age, gender, and ethnicity. In addition, the primary retinal diagnosis, along with a list of other ocular problems addressed or observed during that visit by the retinal specialist was collected from the electronic health records. If patients were seen on multiple occasions between the 6th of January and 29th of December 2021, only the most recent visit was included in the study.

### Image processing and grading

**Figure 1:**
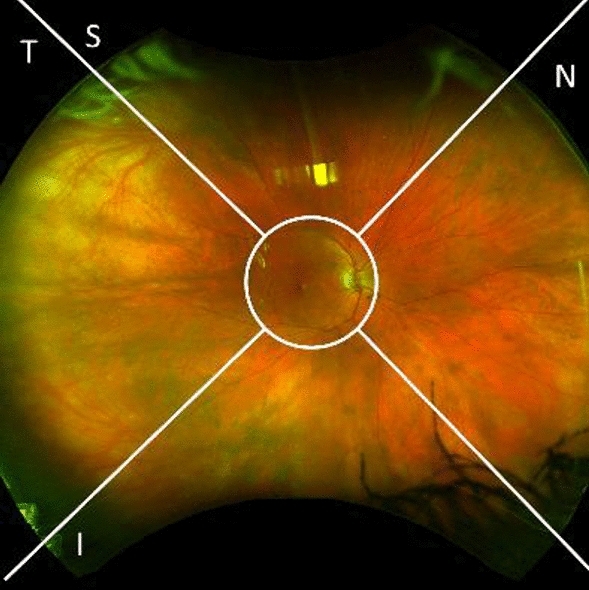
Quadrants utilized in ultra-widefield pseudocolor image evaluation.

The images were directly and securely downloaded from the OptosAdvance database in JPEG format and processed to exclude the central 45° region of the retina, using previously described methods^12^. Each remaining 90° section of the images was then divided into four quadrants: superior (S), nasal (N), inferior (I), and temporal (T) as illustrated in Fig. 1^12^. All images were saved in one document using Microsoft PowerPoint (version 16.58, Microsoft Corporation, Redmond WA) and viewed on the same computer screen. Our analysis only relied on pseudocolor images and did not employ the use of red or green filters.

In order to ensure good image quality for grading, images were required to have the retinal periphery clearly visible and were therefore strictly screened for quality by two independent graders. The imaging inclusion criteria required an unobstructed view of at least 60% of each peripheral quadrant to guarantee substantial visibility, as judged by the graders. If this criterion was fulfilled, each image was then examined for visibility of fourth order arterioles in each quadrant to correct for significant blurriness. Only images that fulfilled both strict criteria for the initial quality check were then forwarded for further analysis (Supplementary Fig. S1) to evaluate abnormalities in the retinal periphery.

A grading system was initially developed for the study by one retinal specialist reviewing 100 retinal images and identifying a comprehensive list of all 41 peripheral abnormalities seen (Supplementary Table S1, Supplementary Fig. S2). To distinguish between distinct peripheral abnormality categories, our retinal specialists agreed on pre-determined definitions of terms that appear to be overlapping. For example, we defined “Pigmentation” to refer to pigmentary abnormalities that were solitary and did not appear to cover a continuous portion of the retinal surface. “Pigmentary degeneration” referred to pigmentation that was in a contiguous pattern along the far periphery whereas “Peripheral degeneration” referred to broader atrophy in the retinal periphery. In addition, “Venous abnormality” encompassed any broad venous changes that were not “Arteriovenous collaterals,” “Collateral Vessels,” “Tortuous vessels,” “Sclerosis of vessels,” or “Venous sheathing”. Similarly, “Intraretinal hemorrhages” was utilized as an umbrella term to describe retinal hemorrhages that were not encompassed by the “dot,” “blot,” or “flame-shaped” hemorrhage categories. One example of each of the abnormalities observed in the study is illustrated in Supplementary Fig. S3.

### Grading validation

In order to validate our grading scheme, an inter-observer validation was first performed. The primary masked retinal specialist and a secondary masked retinal specialist recorded abnormalities found in the periphery of each quadrant across a sample of 100 patient images, using the list of potential abnormalities and being masked to the clinical findings. An extension of Cohen’s Kappa was used to adjust for multi-level comparisons between two raters by deriving proportions of observed and chance agreement with the use of contingency tables. We compared agreement between the graders’ findings on a per quadrant level. Grading disagreements were resolved through the involvement of a senior masked retinal specialist.

Additionally, we utilized a test–retest validation to assess reliability for one our masked retinal specialists. The abnormalities found in the periphery of each quadrant were recorded for 100 patient images at two different time points by the same retinal specialist, 1 week apart. The adjusted Cohen’s Kappa method for multi-level comparisons was utilized again to assess the test–retest reliability.

### Statistical analysis

Subject-level demographic and clinical characteristics are presented as count (%) and mean (95% CI) for categorical and continuous measurements, respectively. To examine the presence of relationships between abnormalities recorded by the masked retinal specialist and the primary retinal diagnoses of the right eye of patients during their visit, two proportion Z-Tests were used. Two-tailed proportion testing allowed us to reject the notion that certain abnormal findings were equally prevalent across the four quadrants. The two-proportion Z-test test was used to find statistically significant proportions of peripheral retinal abnormalities between different quadrants.

Logistic Regression models were utilized to predict the binary presence of abnormal quadrant findings using the independent covariates Age, Sex, Race, and Primary Retinal Diagnosis. Our data set was formatted at a per subject-level. Logistic regression models gave insight towards the relationships between each Retinal Diagnosis and abnormal quadrant findings, while adjusting for subject demographic information. However, the Fisher’s Exact Test was used to further test for significant associations that the Logistic Regression models may have been unable to address, especially among abnormalities with substantially smaller sample sizes in our data set. Pearson correlation coefficients and Point-biserial correlation coefficients were utilized to determine the strength of linear associations between each abnormal quadrant finding and subject demographic information. Statistical analysis was performed using the R statistical programming software Version 4.2.0^15^. Statistical significance was defined as a p-value less than 0.05.

## Results

A total of 1671 ultra-widefield right eye pseudocolor images were initially collected from individual consecutive patients seen in clinic during 2021 (6th January–29th December 2021). The patients had a mean age of 62.73 ± 1.3 years. 928 (55.5%) were female with 959 (57.4%) self-identifying as Caucasian, 141 (8.4%) as Hispanic and 254 (15.2%) as Asian illustrated in Table 1. In order to ensure that each quadrant was visible an initial quality check was performed. After initial screening, 971 (58.12%) images passed quality checking. The reasons for screening failure for the 700 (41.88%) patients excluded in the final analysis are summarized in Supplementary Table S2. The age and sex of the patients excluded from the study were not significantly different from those included in the study.

**Table 1:** Patient demographics.

Demographic category	Gradable subjects N = 971	Ungradable subjects N = 700
Age	60.90 (59.77, 62.03)	64.55 (63.08, 66.02)
Race		
American Indian or Alaska Native	6 (0.6%)	6 (0.9%)
Asian	152 (15.7%)	102 (14.6%)
Black or African American	41 (4.2%)	27 (3.9%)
Hispanic	81 (8.3%)	60 (8.6%)
Native Hawaiian or Other Pacific Islander	4 (0.4%)	4 (0.6%)
Other or mixed race	91 (9.4%)	79 (11.3%)
Refuses to declare	35 (3.6%)	24 (3.4%)
White	561 (57.8%)	398 (56.9%)
Sex		
Female	567 (58.4%)	361 (51.6%)
Male	404 (41.6%)	339 (48.4%)

### Grading validation

In order to validate the grading system, an inter-observer agreement was first measured. The results from the inter-observer validation of 100 patient images graded by quadrant using Cohen’s Kappa provided an overall agreement of 76.1% and a Kappa of 0.542 (p < 0.0001). These values indicated a moderate level of inter-observer agreement. To further validate the grading system, we next performed a test–retest study^16^. The results from the Cohen’s Kappa for the test–retest validation using 100 images yielded an overall agreement of 95.1% and a Kappa of 0.677 (p < 0.0001) indicating a substantial level of agreement^16^.

### Prevalence of abnormalities

Of the 971 images included in the final grading analysis, 625 patients (64.4%) had peripheral abnormalities in at least one quadrant. However, 346 patients (35.6%) had no peripheral abnormalities in any quadrant. Abnormalities were most commonly recorded in the temporal quadrant (n = 822, 34.1%), followed by the nasal (n = 633, 26.2%), inferior (n = 546, 22.6%), and superior (n = 411, 17.0%) quadrants, summarized in Table 2.

**Table 2:** Summary of peripheral abnormalities recorded by quadrant.

Finding	Superior	Inferior	Nasal	Temporal	Total (% of total sample)
Drusen	88	110	183	130	511 (52.63%)
Peripheral degeneration	70	131	145	160	506 (52.11%)
Pigmentation	34	57	50	92	233 (24%)
Laser marks	48	53	45	60	206 (21.22%)
Dot hemorrhage	40	36	51	75	202 (20.8%)
Blot hemorrhage	23	22	34	57	136 (14.01%)
Chorioretinal atrophy	22	33	21	43	119 (12.26%)
Tortuous vessels	27	25	28	23	103 (10.61%)
White without pressure	10	13	15	60	98 (10.09%)
Pigmentary degeneration	10	18	18	24	70 (7.21%)
Sclerosis of vessels	8	13	9	27	57 (5.87%)
Venous abnormality	6	6	9	18	39 (4.02%)
Hard exudates	8	8	8	8	32 (3.3%)
Lattice	8	5	4	14	31 (3.19%)
Cobblestone degeneration	2	6	3	8	19 (1.96%)
Retinal tear	3	2	3	5	13 (1.34%)
Retinal detachment	0	3	1	7	11 (1.13%)
Bony spicules	1	1	1	2	5 (0.51%)
Flame-shaped hemorrhage	1	1	3	0	5 (0.51%)
Cotton wool spots	1	1	1	1	4 (0.41%)
Demarcation ridge	1	1	1	1	4 (0.41%)
Horse-shoe tear	0	1	0	1	2 (0.21%)
CHRPE	0	0	0	2	2 (0.21%)
Retinal hole	0	0	0	2	2 (0.21%)
Macroaneurysm	0	0	0	1	1 (0.1%)
Neovascularization	0	0	0	1	1 (0.1%)

The most prevalent of these peripheral abnormalities included drusen (n = 221, 22.76%), peripheral degeneration (n = 185, 19.05%), and pigmentation (n = 146, 15.04%). In addition, the overall prevalence estimated per 1000 retinal clinic patients was calculated in Table 3.

**Table 3:** Summary of peripheral abnormalities noted by subject.

Peripheral abnormalities	Number of subjects with peripheral abnormality	% of total sample	Prevalence of abnormality per 1000 patients
Drusen	221	22.76	227.6
Peripheral degeneration	185	19.05	190.5
Pigmentation	146	15.04	150.4
Dot hemorrhage	95	9.78	97.8
Laser marks	72	7.42	74.2
Blot hemorrhage	69	7.11	71.1
White without pressure	67	6.90	69
Chorioretinal atrophy	61	6.28	62.8
Sclerosis of vessels	32	3.30	33
Tortuous vessels	32	3.30	33
Pigmentary degeneration	27	2.78	27.8
CHRPE	2	0.21	21
Venous abnormality	20	2.06	20.6
Hard exudates	15	1.54	15.4
Lattice	15	1.54	15.4
Retinal tear	10	1.03	10.3
Cotton wool spots	1	0.10	10
Cobblestone degeneration	9	0.93	9.3
Retinal detachment	7	0.72	7.2
Flame shaped hemorrhage	3	0.31	3.1
Bony spicules	2	0.21	2.1
Retinal hole	2	0.21	2.1
Horseshoe tear	2	0.21	2.1
Macroaneurysm	1	0.10	1
Neovascularization	1	0.10	1
Demarcation ridge	1	0.10	1

When comparing peripheral abnormalities observed on a per quadrant basis using the Chi-Squared Two-proportion test, drusen was observed most commonly in the nasal quadrant with the inferior and temporal quadrants (p < 0.001). Additionally peripheral degeneration was found to be least prevalent in the superior quadrant when compared to the other quadrants (p < 0.001). Other significant findings by quadrant were observed in chorioretinal atrophy (p = 0.01), white without pressure (p < 0.001), and pigmentation (p < 0.001) occurring most prevalently in the temporal quadrant. Retinal detachment was also noted to be more commonly seen in the superior quadrant (p = 0.02).

### Associations of peripheral retinal abnormalities with disease

To understand whether any peripheral retinal abnormalities were associated with disease, we initially performed a logistic regression analysis. These models predicted abnormal occurrence utilizing the independent variables of age or retinal diagnoses.

These logistic regression models identified significant associations between known associations including DR and blot hemorrhages (p < 0.001) and dot hemorrhages (p < 0.001), while retinal vein occlusions were associated with sclerosis of vessels (p < 0.001), tortuous vessels (p < 0.001) and blot hemorrhages (p < 0.001). Additionally, age was associated with peripheral drusen (p < 0.001) which has been described previously^7,8^. This further helped validate the grading system and gradings by the retinal specialists.

In instances when the sample size was reduced, we utilized the Fisher Test to find any additional associations between retinal peripheral abnormalities and retinal diseases. These models also resulted in numerous statistically significant relationships between peripheral abnormalities and retinal disease. Peripheral drusen was associated with AMD (p < 0.001). In addition, DR was also associated with hard exudates (p < 0.001), sclerosis of vessels (p < 0.001), and laser marks (p < 0.001). Again, these associations are already known and further validate the grading.

The grading and logistic regression analysis also revealed some unexpected associations even after accounting for differences in age, particularly in cases of DR. These included strong associations between DR and peripheral drusen (p = 0.004) and chorioretinal atrophy (p = 0.008) and a weaker association with peripheral pigmentation (p = 0.041) suggesting associations between peripheral outer retinal atrophic processes and DR.

All statistically significant associations that resulted from both the logistic regression models and Fisher tests are further summarized in Table 4. Although these statistically significant p-values indicate relationships between retinal diseases and peripheral retinal findings, they do not quantify the strength of their association. Taken together, these results demonstrate that peripheral retinal findings are abundant in retinal patients and vary in prevalence. There appear to be strong statistically significant relationships between some peripheral abnormalities and retinal diseases.

**Table 4:** Summary of statistically significant p-values found using the logistic regression models and Fisher Exact Test.

Independent variable	Peripheral abnormality	Logarithmic P-value	Fisher test P-value
Age	Chorioretinal atrophy	0.001	–
	Drusen	< 0.001	0.001
	Laser marks	0.011	–
	Lattice	–	0.003
	Peripheral degeneration	< 0.001	0.001
	Retinal detachment	0.012	0.002
	White without pressure	0.024	0.002
Age related macular degeneration (AMD)	Blot hemorrhage	–	0.002
	Cobblestone degeneration	–	0.003
	Dot hemorrhage	–	0.001
	Drusen	–	< 0.001
	Laser marks	–	0.005
	Peripheral degeneration	–	< 0.001
	Pigmentary degeneration	–	0.001
Diabetic retinopathy (DR)	Blot hemorrhage	< 0.001	< 0.001
	Chorioretinal atrophy	0.008	–
	Dot hemorrhage	< 0.001	< 0.001
	Drusen	0.004	< 0.001
	Hard exudates	–	< 0.001
	Laser marks	0.001	< 0.001
	Peripheral degeneration	–	0.02
	Pigmentation	0.041	–
	Pigmentary degeneration	–	0.023
	Sclerosis of vessels	–	< 0.001
	Venous abnormality	–	0.008
Diabetes without retinopathy	Blot hemorrhage	–	0.001
	Chorioretinal atrophy	–	0.004
	Dot hemorrhage	–	< 0.001
	Laser marks	–	< 0.001
	Pigmentation	0.034	–
	Sclerosis of vessels	–	0.037
Drusen	Peripheral degeneration	–	0.015
Myopia	Chorioretinal atrophy	< 0.001	–
Nuclear sclerosis	White without pressure	–	0.024
Plaquenil	Drusen	–	0.038
	Pigmentation	–	0.006
Posterior vitreous detachment (PVD)	Dot hemorrhage	–	< 0.001
	Drusen	0.031	–
	Peripheral degeneration	0.017	–
Retinal detachment	Laser marks	0.004	0.005
	Retinal detachment	–	< 0.001
Retinal tear	Chorioretinal atrophy	< 0.001	< 0.001
	Laser marks	0.001	0.005
	Pigmentation	–	0.013
	Retinal tear	–	< 0.001
Retinal vein occlusion	Blot hemorrhage	0.002	–
	Dot hemorrhage	< 0.001	< 0.001
	Sclerosis of vessels	< 0.001	< 0.001
	Tortuous vessels	0.015	–
	Venous abnormality	0.045	0.016

## Discussion

Examination of pseudocolor ultra-widefield fundus images in our cohort illustrated that peripheral retinal abnormalities are common amongst retinal patients, with peripheral drusen the most prevalent peripheral retinal finding. The identification of abnormalities was validated by findings of associations between diagnosis and peripheral findings previously reported in the literature. We also observed that the grading system for identifying retinal abnormalities was repeatable and could be used reliably between retinal specialists to identify periphery abnormalities on pseudocolor ultra-widefield retinal imaging.

There have been some previous attempts to grade all types of peripheral findings using ultra-widefield retinal imaging. Studies have worked to examine peripheral abnormalities by dividing the retina into novel grids centered on the macula to distinguish changes in patients with AMD^11^. This paper created a novel grid to improve visualization of retinal changes such as drusen, retinal pigment epithelium changes, and overall atrophy in patients with AMD^11^. By contrast, our study hoped to create a broad list of common peripheral retinal abnormalities enabling a grading system that can diagnose a wider range of retinal peripheral changes. We also developed a basic image library to compare and contrast common peripheral abnormalities in the retina to improve the identification and investigation of such characteristics in a clinical setting.

In addition, other studies have looked into dividing the periphery into the standard Early Treatment Diabetic Retinopathy Study (ETDRS) seven standard fields to evaluate vascular changes in patients with DR^14^. This study focused more on how different retinal visualization methods, including ultra-widefield imaging and angiography, can allow for improved visualization of peripheral retinal nonperfusion, vascular leakage and neovascularization in DR patients compared to the ETDRS fields^14^. While we do observe peripheral changes in patients with DR that include some vascular changes such as sclerosis of vessels, we utilized a more general model based on studies identifying peripheral changes in autofluorescence to evaluate a greater variety of peripheral changes using standardized quadrants^12^. Our novel validated grading system includes the observation of peripheral vascular changes in patients with DR with other widespread peripheral retinal abnormalities present in retinal patients.

Looking firstly at peripheral findings, which are known to be associated with specific retinal diseases, we initially focus on DR. In our study, blot hemorrhages, dot hemorrhages, and laser marks were identified to be associated with DR using the logistic regression model and hard exudates, which were seen more rarely, were another peripheral abnormality associated with DR using the Fisher’s exact test. DR is already known to be associated with these peripheral findings which has been reported in numerous other studies^4–6^. Additionally, DR was significantly associated with sclerosis of vessels and venous abnormalities. Sclerosis and venous abnormalities are already known to be associated with diabetic retinopathy using other imaging and exam modalities^17–20^. These findings help to validate our grading system.

Our study also found a significant association between AMD and peripheral drusen and peripheral degeneration. This is similar to the findings of a few previous studies which indicate that peripheral drusen commonly appears in AMD^7–9,21^. While peripheral abnormalities are highly prevalent in patients with AMD, the significance of these findings and implications on vision are not completely understood as such findings imply that AMD is a pan-retinal disease^22^. Our data supports this claim, as patients with AMD in the present study were observed with a significant presence of a variety of peripheral retinal abnormalities, such as blot and dot hemorrhages, and laser marks. Such relationships between AMD and these retinal abnormalities are novel and could warrant further investigation in future studies.

In addition to previously reported relationships, some other relationships identified in this study do not appear to have been reported previously. Chorioretinal atrophy and peripheral drusen were associated with DR. It is unclear why this relationship was found, but it could suggest that as well as inner retinal perfusion, commonly associated with diabetic retinopathy, there may be outer retinal perfusion changes in the periphery of the choroid. Some studies have observed that patients with DR have significant changes in the thickness of the choroid and that choroidal vascular abnormalities are commonly noted in more severe DR, which we did not examine in this study^23,24^. This could be investigated further with peripheral optical coherence tomography angiography (OCT-A)^16–18^.

It is important to note, however, that while ultra-widefield pseudocolor fundus imaging is an important diagnostic tool for some conditions, it does have diagnostic limitations for some retinal conditions. For example, past studies have shown that ultra-widefield pseudocolor only shows a moderate specificity and sensitivity when used to identify peripheral retinal abnormalities such as retinal tears and holes^25,26^. Such limitations can be overcome through the use of various diagnostic techniques such as the continued practice of 360-degree scleral depressed exams and routine OCT-A scans^25,26^. Such multimodal approaches in clinical settings will further improve our understanding of both retinal and systemic diseases. Previous studies have shown that utilizing multimodal retinal imaging such as ultra-widefield pseudocolor fundus imaging with OCT-A scans can improve our ability to understand retinal conditions like diabetic retinopathy and retinal non-perfusion^27–29^. Understanding such outcomes will further shed light on systemic effects that diabetes mellitus has on patients and further supports the need for continued exploration of the many uses of ultra-widefield pseudocolor fundus imaging for other systemic conditions.

Lastly, continuing to gather detailed data about peripheral retinal abnormalities visualized in ultra-widefield pseudocolor images will enable us to develop novel methods to evaluate retinal problems. We are in the beginning stages of understanding the many ways that deep learning and artificial intelligence can be utilized to evaluate retinal abnormalities such as non-perfusion^30^. Our study adds to literature to exploring novel ways that ultra-widefield pseudocolor imaging can be utilized to generate data for training artificial intelligence to evaluate clinical images with greater sensitivity and specificity.

The findings of the study are limited by the retrospective nature of data collection, with an expected large number of images excluded from analysis due to an obscured retinal periphery. To be more confident that prevalence findings, and the associations with retinal disease are true, and in order to reduce the chance of misdiagnosis, a prospective study, with full clinical grading, would be useful to prevent associations with an incorrect retinal diagnosis. The present study would also benefit from replication of the study at other retinal centers to examine and quantify retinal abnormalities utilizing these methods. The study would also be further strengthened through the inclusion of cohorts who are not retinal patients, so that the findings can apply to a broader base of eye patients. However, reassuringly, the present study does validate itself, in numerous ways confirming previously known associations between peripheral retinal findings and retinal disease. In future studies, it would be interesting to focus on cohorts of patients with diagnoses with novel relationships. For instance, studying larger cohorts of DR patients to confirm that peripheral drusen, chorioretinal atrophy and pigmentary degeneration is associated with DR and to understand the strength of this association with stage of disease.

In summary, this study describes a validated peripheral grading system for ultra-widefield pseudocolor retinal imaging and reports the prevalence of retinal abnormalities identified using retinal ultra-widefield imaging in a cohort of retinal clinic patients. The study also identifies some potential associations between disease and peripheral retinal findings which would be of interest for further study. This information is useful for retinal specialists, using ultra-widefield imaging, to gain an understanding of the prevalence of peripheral abnormality and may be of interest to researchers studying biomarkers for disease. Overall, this paper adds to the literature on peripheral findings associated with retinal disease and aims to improve methods of standardization for the use of ultra-widefield pseudocolor fundus imaging in a clinical setting.

### Supplementary Information

Supplementary Information.

## Data Availability

All relevant data is listed in this manuscript. Additional inquiries can be directed towards the corresponding author.

## References

[1] Nagiel A, Lalane RA, Sadda SR, Schwartz SD (2016). Ultra-widefield fundus imaging a review of clinical applications and future trends. RETINA.

[2] Kumar V (2021). Ultra-wide field retinal imaging: A wider clinical perspective. Indian J. Ophthalmol..

[3] Patel SN, Shi A, Wibbelsman TD, Klufas MA (2020). Ultra-widefield retinal imaging: An update on recent advances. Ther. Adv. Ophthalmol..

[4] Tahir, F., Akram, M. U., Abbass, M. & Khan, A. A. Laser marks detection from fundus images. In *2014 14th International Conference on Hybrid Intelligent Systems* 147–151 (2014). 10.1109/HIS.2014.7086188.

[5] Jitpakdee, P., Aimmanee, P. & Uyyanonvara, B. A survey on hemorrhage detection in diabetic retinopathy retinal images. In *2012 9th International Conference on Electrical Engineering/Electronics, Computer, Telecommunications and Information Technology, ECTI-CON 2012* (2012). 10.1109/ECTICon.2012.6254356.

[6] Tjandrasa, H., Putra, R. E., Wijaya, A. Y. & Arieshanti, I. Classification of non-proliferative diabetic retinopathy based on hard exudates using soft margin SVM. In *Proceedings-2013 IEEE International Conference on Control System, Computing and Engineering, ICCSCE 2013* 376–380 (2013) 10.1109/ICCSCE.2013.6719993.

[7] Mullins, R. F., Russell, S. R., Anderson, D. H. & Hageman, G. S. *Drusen associated with aging and age-related macular degeneration contain proteins common to extracellular deposits associated with atherosclerosis, elastosis, amyloidosis, and dense deposit disease*. 10.1096/fasebj.14.7.835. 10783137

[8] Khan KN (2016). Differentiating drusen: Drusen and drusen-like appearances associated with ageing, age-related macular degeneration, inherited eye disease and other pathological processes. Prog. Retin. Eye Res..

[9] Domalpally A (2017). Peripheral retinal changes associated with age-related macular degeneration in the age-related eye disease study 2: Age-related eye disease study 2 report number 12 by the age-related eye disease study 2 Optos PEripheral RetinA (OPERA) Study Research Group. Ophthalmology.

[10] del Priore LV, Kaplan HJ, Tezel TH (2008). Therapeutic agents for posterior segment vitrectomy surgery. Ocular Ther..

[11] Oellers P (2017). Novel grid combined with peripheral distortion correction for ultra-widefield image grading of age-related macular degeneration. Clin. Ophthalmol..

[12] Suetsugu T (2016). Evaluation of peripheral fundus autofluorescence in eyes with wet age-related macular degeneration. Clin. Ophthalmol..

[13] Nagiel A, Lalane RA, Sadda SR, Schwartz SD (2016). Ultra-widefield fundus imaging: A review of clinical applications and future trends. Retina.

[14] Soliman AZ, Silva PS, Aiello LP, Sun JK (2012). Ultra-wide field retinal imaging in detection, classification, and management of diabetic retinopathy. Semin. Ophthalmol..

[15] R Core Team. *R: A Language and Environment for Statistical Computing*. Preprint at (2021).

[16] McHugh ML (2012). Interrater reliability: The kappa statistic. Biochem. Med..

[17] Ashton N (1949). Vascular changes in diabetes with particular reference to the retina vessels. Br. J. Opthalmol..

[18] Nguyen, T. T. & Wong, T. Y. *Retinal Vascular Changes and Diabetic Retinopathy* (2009). 10.1007/s11892-009-0043-4 19640340

[19] Klein R, Klein BEK, Moss SE, Wong TY (2007). Retinal vessel caliber and microvascular and macrovascular disease in type 2 diabetes: XXI: The Wisconsin Epidemiologic Study of Diabetic Retinopathy. Ophthalmology.

[20] Kalaw FGP, Sharma P, Kako RN, Walker E, Borooah S (2023). Peripheral retinal vessel whitening in patients with diabetes mellitus. Sci. Rep..

[21] Buschini E, Piras A, Nuzzi R, Vercelli A (2011). Age related macular degeneration and drusen: Neuroinflammation in the retina. Prog. Neurobiol..

[22] Forshaw TRJ, Minör ÅS, Subhi Y, Sørensen TL (2019). Peripheral retinal lesions in eyes with age-related macular degeneration using ultra-widefield imaging: A systematic review with meta-analyses. Ophthalmol. Retina.

[23] Wang H, Tao Y (2019). Choroidal structural changes correlate with severity of diabetic retinopathy in diabetes mellitus. BMC Ophthalmol..

[24] Hamadneh T (2020). Choroidal changes in diabetic patients with different stages of diabetic retinopathy. Cureus.

[25] Lin AC (2023). The sensitivity of ultra-widefield fundus photography versus scleral depressed examination for detection of retinal horsehoe tears. Am. J. Ophthalmol..

[26] Khan M (2023). Evaluating Ultra-widefield Imaging Utility in the Detection of Treatment-requiring Peripheral Retinal Tears and Holes. Retina.

[27] Vujosevic S (2023). Multimodal retinal imaging in patients with diabetes mellitus and association with cerebrovascular disease. Ophthalmic Res..

[28] Ashraf M, Hock KM, Cavallerano JD, Wang FL, Silva PS (2022). Comparison of widefield laser ophthalmoscopy and ETDRS retinal area for diabetic retinopathy. Ophthalmol. Sci..

[29] Stino H (2023). Association of diabetic lesions and retinal nonperfusion using widefield multimodal imaging. Ophthalmol. Retina.

[30] Inoda S (2022). Deep-learning-based AI for evaluating estimated nonperfusion areas requiring further examination in ultra-widefield fundus images. Sci. Rep..

