# Supplementary material for: Prevalence of peripheral retinal findings in retinal patients using ultra-widefield pseudocolor fundus imaging
Source: Sci Rep. 2023 Nov 22;13:20515. doi: 10.1038/s41598-023-47761-x (PMC10665364; doi:10.1038/s41598-023-47761-x)
Supplement: Supplementary file 1 — Supplementary Information. [file 41598_2023_47761_MOESM1_ESM.pdf]

Supplementary Figure S1. **Representative images with decisions after initial quality control.** Images 1-3 were excluded from final analysis based on the unfulfilled quality criteria. Image 1 lacked 60% visibility of each quadrant and the visibility of at least fourth-order arteriole count in each quadrant. Image 2 lacked 60% visibility of each quadrant. Image 3 lacked a minimum visibility of fourth-order arterioles in each quadrant. Image 4 fulfilled both the quality criteria and was forwarded for further analysis.

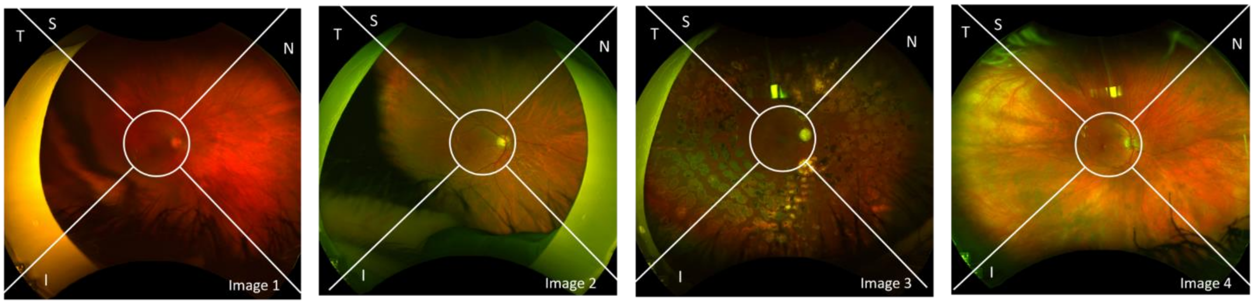

Supplementary Figure S2. Examples of peripheral retinal abnormalities identified on ultra-widefield color fundus imaging. Image 1 is taken from a 54-year-old male patient diagnosed with DR. Image 2 is taken from a 93-year-old female patient diagnosed with AMD.

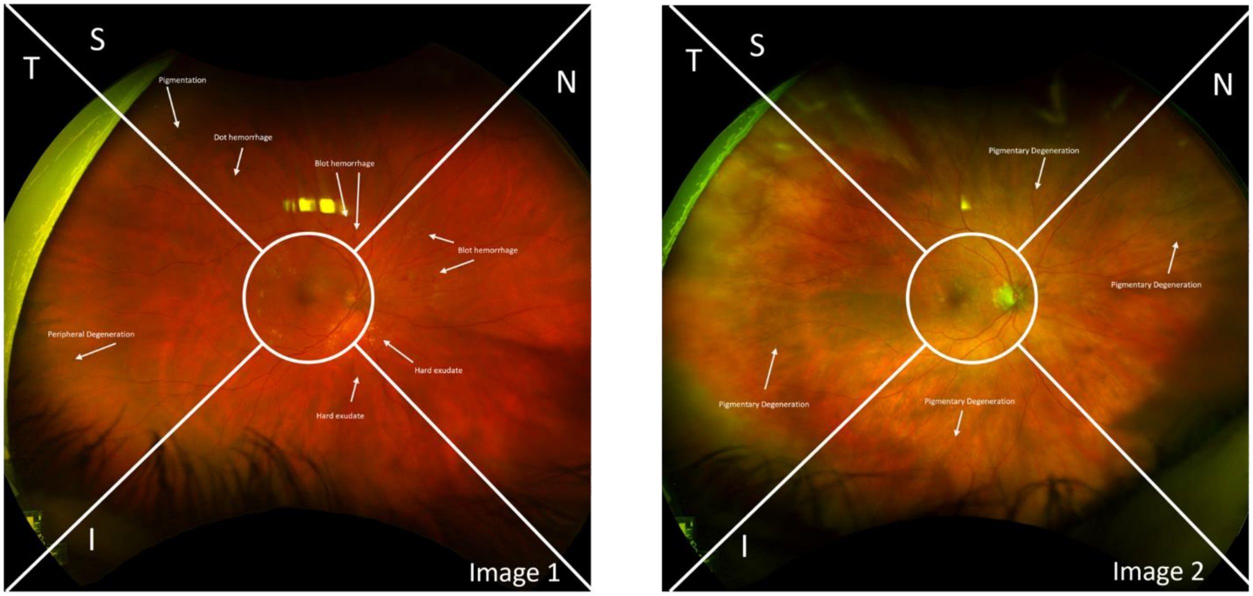

Supplementary Figure S3. Example illustrations of each peripheral abnormality identified in the study. These images illustrate the abnormality examples used by the masked retinal specialists to systematically evaluate peripheral abnormalities by quadrant in this study.

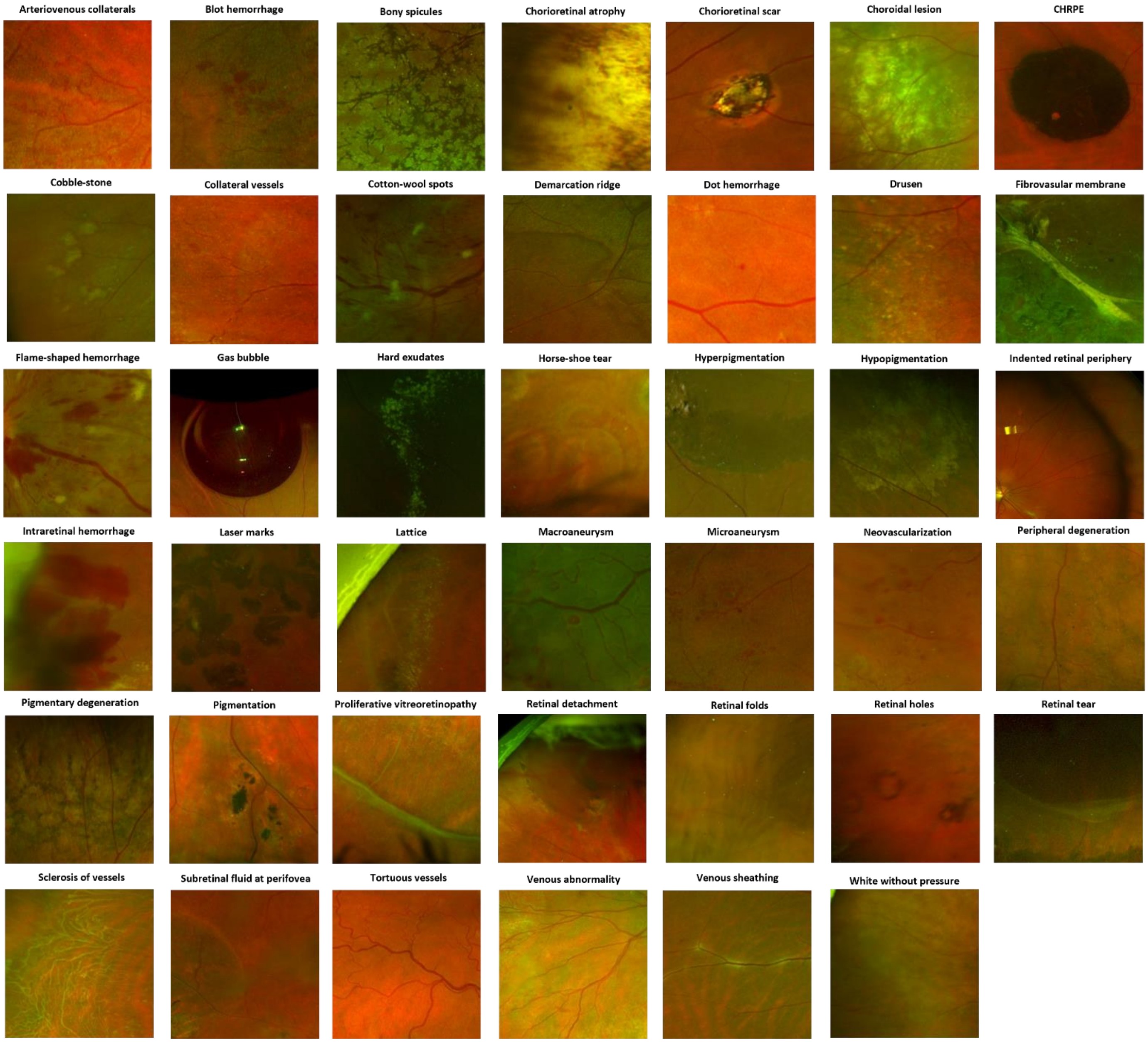

Supplementary Table S1. List of retinal abnormalities from the first 100 images graded. This table illustrates the abnormality list supplied to the masked retinal specialists to systematically evaluate peripheral abnormalities by quadrant in this study.

Comprehensive Peripheral Abnormalities List
Arteriovenous Collaterals
Blot Hemorrhage
Bony Spicules
Chorioretinal Atrophy
Chorioretinal Scar
Choroidal Lesion
CHRPE
Cobble Stone
Collateral Vessels
Cotton Wool Spots
Demarcation Ridge
Dot Hemorrhage
Drusen
Fibrovascular Membrane
Flame-Shaped Hemorrhage
Gas bubble
Hard Exudates
Horse-Shoe Tear
Hyperpigmentation
Hypopigmentation (Hypopigmentation membrane)
Indented Retinal Periphery
Intraretinal Hemorrhage
Laser Marks
Lattice
Macroaneurysm
Microaneurysm
Neovascularization
Peripheral Degeneration
Pigmentary Degeneration
Pigmentation
Proliferative Vitreoretinopathy
Retinal Detachment
Retinal Folds
Retinal Hole
Retinal Tear
Sclerosis Of Vessels
Subretinal Fluid at the Perifovea
Tortuous Vessels
Venous Abnormality
Venous Sheathing
White Without Pressure

Supplementary Table 2. Summary of images which failed initial quality check.

Quality Screening Criteria	Ungradable Subjects N = 700
**Superior Quadrant Visibility**	
**Less Than 60%**	282 (40.3%)
**Greater 60%**	418 (59.7%)
**Nasal Quadrant Visibility**	
**Less Than 60%**	160 (22.9%)
**Greater 60%**	540 (77.1%)
**Inferior Quadrant Visibility**	
**Less Than 60%**	435 (62.1%)
**Greater 60%**	265 (37.9%)
**Temporal Quadrant Visibility**	
**Less Than 60%**	286 (40.9%)
**Greater 60%**	414 (59.1%)
**Superior Arterioles**	
**Less Than 4 Arterioles Visible**	77 (85.6%)
**4+ Arterioles Visible**	13 (14.4%)
**Nasal Arterioles**	
**Less Than 4 Arterioles Visible**	68 (75.6%)
**4+ Arterioles Visible**	22 (24.4%)
**Inferior Arterioles**	
**Less Than 4 Arterioles Visible**	72 (80.0%)
**4+ Arterioles Visible**	18 (20.0%)
**Temporal Arterioles**	
**Less Than 4 Arterioles Visible**	54 (61.4%)
**4+ Arterioles Visible**	34 (38.6%)
